# New dosing schedules of dasatinib for CML and adverse event management

**DOI:** 10.1186/1756-8722-2-10

**Published:** 2009-02-23

**Authors:** Siu-Fun Wong

**Affiliations:** 1 Western University of Health Sciences, College of Pharmacy, Pomona, CA, USA

## Abstract

Resistance to imatinib in patients with chronic myelogenous leukemia (CML) or Philadelphia chromosome-positive acute lymphoblastic leukemia (Ph+ ALL) has emerged as a significant clinical issue. Dasatinib is a tyrosine kinase inhibitor that has 325-fold greater in vitro activity against native BCR-ABL (breakpoint cluster region-Abelson leukemia virus) compared with imatinib and can overcome primary (intrinsic) and secondary (acquired) imatinib resistance. Here, we review the clinical profile of dasatinib in imatinib-resistant and -intolerant patients and share clinical approaches for managing adverse events (AEs) to ensure maximum patient benefit. References were obtained through literature searches on PubMed as well as from the Proceedings of Annual Meetings of the American Society of Clinical Oncology, the American Society of Hematology, and European Hematology Association. Phase II and III studies of dasatinib in patients with imatinib-resistant or -intolerant CML in any phase or Ph+ ALL were selected for discussion. Dasatinib is currently indicated for the treatment of patients with imatinib-resistant or -intolerant CML or Ph+ ALL. AEs associated with dasatinib are typically mild to moderate, and are usually resolved with temporary treatment interruption and/or dose adjustments. A Phase III dose optimization study showed that in patients with chronic phase (CP) CML, 100 mg once-daily dasatinib improves the safety profile, particularly pleural effusion and thrombocytopenia, while maintaining efficacy compared with the previously recommended dose of 70 mg twice-daily. Dasatinib has a manageable safety profile. For patients with CP CML, a new recommended starting dose of 100 mg once daily has recently been approved. The recommended dose for patients with advanced CML or Ph+ ALL remains 70 mg twice daily.

## Introduction

Chronic myelogenous leukemia (CML) is a myeloproliferative disorder of blood stem cells [1]. It accounts for approximately 15% of all leukemias diagnosed in adults [2]. The causative molecular defect is the BCR-ABL protein, which is encoded by the Philadelphia chromosome (Ph) [3]. This genetic aberration arises from an exchange of genetic material between chromosomes 9 and 22, which results in the fusion of the breakpoint cluster region (*BCR*) and the Abelson leukemia virus (*ABL*) genes. The resulting gene encodes a protein kinase that is constitutively active and activates a number of proteins involved in regulating the cell cycle. Cell division is accelerated and DNA repair is affected.

The median age of patients at the time of CML presentation is 53 years; however, the disease can affect patients of all ages [1]. CML typically begins with a chronic phase and, over the course of 3 to 5 years, progresses from an accelerated phase (AP) to a blast crisis (BC) or terminal phase [1]. Approximately 85% of patients with CML are diagnosed in the chronic phase and are usually asymptomatic or have only mild symptoms [4]. Some of the criteria used to define the accelerated phase are more than 20% basophils and 10% to 19% myeloblasts in the blood or bone marrow, cytogenetic evolution with new abnormalities in addition to the Ph chromosome, and increased white blood cell count unresponsive to therapy [4]. The accelerated phase is significant because it signals that the disease is progressing and that transformation to the blast phase is imminent. The blast phase is most often the final phase of CML, behaving like acute leukemia, with rapid progression and short survival of the patients. Characteristics include more than 20% myeloblasts or lymphoblasts in the blood or bone marrow, large clusters of blasts in the bone marrow on biopsy, and development of a solid focus of leukemia outside the bone marrow [4,5].

Ph is also the most common genetic abnormality in adult acute lymphocytic leukemia (ALL); approximately 25% of adult cases are Ph+ [6,7]. Furthermore, the incidence of Ph+ ALL increases with age, accounting for an estimated 50% of ALL cases in patients older than 50 years [6,7]. Patients with Ph+ ALL progress rapidly and are at high risk of developing central nervous system leukemia [8], highlighting the need for improved treatments.

The identification of the BCR-ABL kinase fusion protein and its role in the pathogenesis of CML has led to the emergence of BCR-ABL-targeted therapy, which has become the standard of care for patients with CML. Historically, patients with CML were treated with agents such as hydroxyurea, cytarabine, and alfa-interferon. Although hydroxyurea improves outcomes compared with previous chemotherapies, it has a transient effect and was superseded by the use of alfa-interferon and, subsequently, combinations of chemotherapy and alfa-interferon [9]. Alfa-interferon treatment significantly prolongs survival but is confounded by associated toxicities [9]. Allogeneic stem cell transplantation following high-dose chemotherapy is a potentially curative treatment for patients with chronic phase CML (CP CML) [1]. However, the success of this procedure is significantly lower in patients older than 40 years because of the procedure's toxicity. Other variables may also influence outcome, such as disease stage and level of donor-recipient human leukocyte antigen (HLA) matching [1].

**Figure 1:**
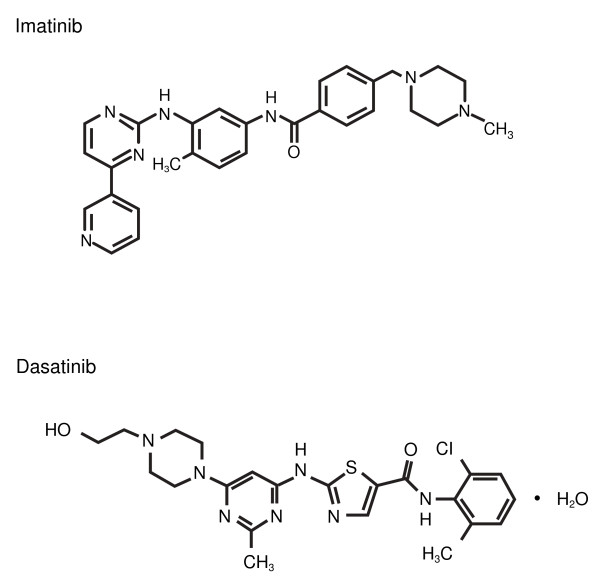
**Chemical structures of imatinib (C_29_H_31_N_7_O) and dasatinib (C_22_H_26_C_1_N_7_O_2_S)**.

The emergence of BCR-ABL-targeted agents has revolutionized the treatment of CML. These agents compete with adenosine triphosphate (ATP) for its binding pocket on the BCR-ABL protein, preventing the functioning of the BCR-ABL enzyme and ultimately killing the cell. In 2001, imatinib became available and was the first BCR-ABL-targeted agent indicated for the treatment of CML; a determination based on the Phase III International Randomized Study of Interferon versus STI571 (IRIS clinical trial) (Figure 1). In this study, imatinib induced greater response rates, improved freedom from progression to AP or BC, and was better tolerated compared with alfa-interferon plus cytarabine [10]. Imatinib has since become the standard first-line therapy for patients with CP CML and is indicated for the treatment of patients with AP or BC CML [11].

Despite the significant improvements in the outcome for patients with CML treated with imatinib [12], resistance to this agent has emerged as a significant clinical issue [13]. Results from a Phase III randomized study have demonstrated that a number of patients have primary or acquired resistance to imatinib treatment [14,15]. In this study, primary resistance developed in an estimated 5% of patients with newly diagnosed CP CML who failed to achieve a complete hematologic response (CHR) with imatinib at 3 months. A further 16% who failed to achieve a major cytogenetic response (MCyR) with imatinib at 12 months, and 24% who failed to achieve a complete cytogenetic response (CCyR) at 18 months [14-16]. Relapse in patients receiving imatinib occurred in approximately 17% of patients over 5 years, and 7% of patients developed disease progression after the same period of time [14]. Resistance to imatinib is more common in patients who are in advanced stages of CML, and relapse occurs in the majority of these patients who initially respond to treatment [17-19]. In a single-center study of patients with BCR-ABL-positive leukemias, the percentages of patients in CP, AP, or blast phase (BP) CML with imatinib resistance were 29%, 45%, and 92%, respectively [18].

Although most imatinib-related adverse events (AEs) generally resolve with dose modifications or interruptions, intolerance is a concern in patients treated with imatinib. After a median follow-up of 4.5 years, 5% of patients in the IRIS trial discontinued imatinib as a result of AEs [20]. Grade 3 or 4 AEs reported in this study included neutropenia, thrombocytopenia, anemia, and elevated liver enzymes [20].

Currently, there are several treatment options available for the second-line treatment of CML. Increasing the dose of imatinib (600–800 mg/day) is one option for treating patients who are resistant to standard doses (400 mg/day). Studies have shown that 35% to 40% of patients with CML who did not respond with standard-dose imatinib subsequently achieved an MCyR with high-dose imatinib [21,22]. However, these responses were short-lived with most patients relapsing within 2 to 11 months. Use of new and more potent tyrosine kinase inhibitors can be a second option; two other BCR-ABL inhibitors are now available for the treatment of patients with imatinib resistance or intolerance. Nilotinib, was introduced in November, 2007 for the treatment of patients with CP or AP CML who are resistant to or intolerant of imatinib. Dasatinib is indicated for the treatment of patients with imatinib-resistant or -intolerant CML in any phase or Ph+ ALL. In this article, we review the clinical profile of dasatinib in imatinib-resistant and -intolerant patients, discuss the newly approved dose for patients with CP CML, and share clinical approaches in managing AEs to ensure maximum patient benefit.

### Dasatinib

Dasatinib is a thiazole carboximide agent with potent activity against the BCR-ABL kinase fusion protein and the v-src sarcoma viral oncogene homolog (SRC) family kinases (SFKs) (Figure 1) [23]. Dasatinib also has activity against other oncogenic tyrosine kinases, such as c-Kit, platelet-derived growth factor-receptor (PDGFR), and ephrin-A receptor [23-26]. In vitro, dasatinib demonstrated 325-fold greater activity against native BCR-ABL compared with imatinib [27,28].

Treatment with dasatinib can overcome BCR-ABL-dependent and -independent mechanisms of resistance to imatinib. Because of the greater potency of dasatinib against native BCR-ABL, dasatinib may have activity in patients with imatinib resistance caused by *BCR-ABL *overexpression [29,30]. Dasatinib can also overcome mechanisms of resistance caused by BCR-ABL kinase mutations and has demonstrated activity against the majority of known imatinib-resistant mutations except T315I, and had shown relative insensitivity to F317l [27,28,31].

BCR-ABL-independent imatinib resistance may develop as a result of activation of alternative signaling pathways. In vitro studies using SRC kinase inhibitors reveal that SFKs play an active role in BCR-ABL signal transduction and have demonstrated that inhibition of SFKs results in growth arrest and induction of apoptosis in Ph+ CML cell lines [32]. Furthermore, one SFK, LYN, was highly overexpressed and activated in bone marrow cell lines established from patients with resistance to imatinib. Inhibition of LYN reduced proliferation and survival of these imatinib-resistant cell lines compared with the nonresistant parental cell population, implicating SFK activation in some instances of imatinib resistance [33]. The inhibition of SFKs by dasatinib may contribute to the efficacy of this agent in BCR-ABL-independent imatinib resistance, helping to improve overall outcome. In the United States and Europe, dasatinib is the first and only dual SFK/BCR-ABL inhibitor indicated for the treatment of imatinib-resistant and imatinib-intolerant patients in all phases of CML or with Ph+ ALL.

### Dosage and administration

Dasatinib was initially indicated for patients with CML in any phase or with Ph+ ALL at a starting dose of 70 mg twice daily. This dose was chosen based on Phase I study data. However, similar efficacy was noted with doses of 100 mg/day or greater, irrespective of the schedule of administration [34]. As a result, a dose optimization study (CA180-034) was launched, and results from this study eventually led to a change in the recommended starting dose for patients with CP CML at100 mg administered orally once daily, either in the morning or in the evening. For patients with AP CML, myeloid BP CML, lymphoid BP CML, or Ph+ ALL, the recommended starting dose remains 70 mg twice daily. Dose modifications may be warranted, depending on tolerability or level of response to dasatinib.

Dasatinib is supplied as white to off-white, film-coated, biconvex tablets (20, 50, and 70 mg) to be stored at room temperature. To minimize the risks of medication error, it is also necessary to caution patients that varying doses of dasatinib are each available in the same color tablets. This is specifically warranted if dose adjustments are required where more than one dose strength of the tablets are needed to deliver the appropriate dose. A patient diary is highly recommended to encourage patient compliance. Tablets should not be crushed or cut, but should be swallowed whole, and can be taken with or without food. If vomiting occurs within 30 minutes after dosing, repeat dosing should be considered.

### Pharmacokinetics

Pharmacokinetic analysis has demonstrated that dasatinib is rapidly absorbed and exhibits linear elimination characteristics. Dasatinib C_max _occurs between 0.5 and 6 hours following administration, and the overall mean terminal half-life is 3 to 5 hours [35]. The cause of the variability in C_max _has not yet been determined; however, it is likely due to the result of inherent differences between the patients studied. The low bioavailability of dasatinib cannot be attributed to P-glycoprotein activity, as dasatinib is not a substrate for this drug transporter [36]. No clinically relevant effects on absorption have been observed from food consumption. In preclinical models, the oral bioavailability of dasatinib ranged from 14% to 34%. In patients, the total volume of distribution of dasatinib is 2505 L, implying that dasatinib is well distributed in extravascular space [35].

The metabolism of dasatinib is mainly hepatic, and the CYP3A4 enzyme is primarily responsible for the formation of active metabolites [35]. The FMO-3 enzyme and UGT enzymes are also involved in the formation of dasatinib metabolites. The active metabolites of dasatinib have been identified as hydroxylated metabolites (M20 and M24), an *N*-dealkylated metabolite (M4), an *N*-oxide metabolite (M5), an acid metabolite (M6), and the glucuronide conjugates (M8a, b) [37]. These metabolites are unlikely to play a major role in the observed pharmacology of the drug, as they were found to represent only about 5% of the area under the plasma drug concentration-time curve (AUC). Dasatinib is primarily excreted as metabolites (85%) in the feces [37].

There are no clinically relevant effects of age and gender on the pharmacokinetics of dasatinib, although dasatinib has not been evaluated in pediatric patients and is therefore not recommended as a treatment option for this patient population. There are currently no clinical studies conducted in patients with impaired liver or renal function. The metabolism of dasatinib is mainly hepatic via oxidative biotransformation; therefore, caution is recommended in patients with hepatic impairment. The percentage of dasatinib and its metabolites that is excreted via the kidney is less than 4% [35,37].

### Drug interactions

Dasatinib is a CYP3A4 substrate. Studies have shown that the AUC and/or C_max _of dasatinib are altered when coadministered with ketoconazole (a CYP3A4 inhibitor), simvastatin (a CYP3A4 substrate/inhibitor) or rifampin (a CYP3A4 inducer) [38]. Therefore, use of CYP3A4 substrates/inhibitors during dasatinib treatment should be avoided if possible, as this may increase bioavailability of dasatinib. If use of CYP3A4 inhibitors cannot be avoided, patients should be monitored closely for dasatinib toxicity and reduction of the dose by 20 to 40 mg per day should be considered. CYP3A4 inducers may decrease dasatinib plasma concentrations. In patients who require CYP3A4 inducers, alternate agents with less enzyme induction potential should be used if possible. If the use of a CYP3A4 inducer is necessary, dasatinib dose escalation in 20 mg increments is recommended. St. John's wort, a CYP3A4 inducer, may also decrease dasatinib plasma concentrations and should not be taken by patients receiving dasatinib (Additional file 1, Table S1) [11,35,39].

Preclinical data have shown that the solubility of dasatinib is pH-dependent, which suggests that concomitant administration of antacids could decrease the absorption of dasatinib. In a clinical study evaluating the pharmacokinetics of dasatinib, a 55% decrease in AUC was observed when dasatinib was administered simultaneously with antacids. However, no decrease in AUC was reported when antacids were administered 2 hours prior to dasatinib [39]; therefore, antacids should be taken at least 2 hours before or after dasatinib administration. The use of proton pump inhibitors and histamine-2 (H_2_) blockers should be avoided, because such use is likely to decrease the bioavailability of dasatinib for up to 10 hours. The use of antacids in place of proton pump inhibitors or H_2 _blockers is recommended [11,35].

Initial in vitro study data suggested that dasatinib has the potential to prolong cardiac ventricular repolarization (QT interval). In single-arm clinical studies, 3 patients (< 1%) experienced a QTcF > 500 msec. Dasatinib should therefore be administered with caution in patients with a history of QT prolongation, electrolyte imbalance (potassium and magnesium), congenital long QT syndrome, or those receiving concurrent anti-arrhythmic medications, or other medications that can lead to QT prolongation (Additional file 1, Table S1) [35].

### Special populations

Dasatinib use should be avoided in women who are pregnant or considering becoming pregnant. Patients who use dasatinib while pregnant or who become pregnant during dasatinib use should be informed of the potential toxicity to the fetus. Women should not breast-feed while taking dasatinib, because it is unknown whether dasatinib is excreted in human milk [35].

A recently reported set of case studies from clinical trials and post-marketing reports evaluated a small number of patients (8 male and 8 female) who had either conceived or had a partner conceive whilst receiving dasatinib [40]. Of the eight females who became pregnant whilst receiving dasatinib, there were three cases of induced abortion (two due to patient decision, one for unknown reasons), two cases of spontaneous abortion (one at 8 weeks in a 38-year-old patient [G1P1], one at 9 weeks gestation in a 33-year-old [G3P3]) and three deliveries (one normal, one by caesarean and one unknown). Of the eight males with pregnant partners, seven normal newborns were reported, and one case of unknown outcome. The authors concluded that dasatinib treatment does not have a negative impact on pregnancy (for mother or fetus); however, patients receiving dasatinib should be advised to practice adequate contraception.

A larger study evaluated the effect of imatinib on 180 women, with outcomes data available for 125 patients [41]. In total, 50% delivered normal infants, 28% underwent induced abortions (3 due to abnormalities). Abnormalities were identified in 12 infants, with 3 having strikingly similar complex malformations. The authors noted that this was a cause for concern, concluding that whilst most pregnancies have a successful outcome, there appears to be a risk that imatinib exposure may cause malformation.

In clinical studies, no differences in the safety and efficacy of dasatinib were reported between the 23% (119/511) of patients who were older than 65 years and those who were younger (18–65 years). However, it remains possible that some elderly patients may have greater sensitivity to dasatinib. The safety and efficacy of dasatinib have not been established in patients younger than 18 years [35].

### Clinical studies

#### The phase II START program

Dasatinib has shown efficacy in a series of multicenter, open-label Phase II clinical studies with patients in all phases of CML or with Ph+ ALL who are resistant to or intolerant of imatinib (the START [SRC/Abl Tyrosine kinase inhibition Activity: Research Trials of dasatinib] program). Hematologic and cytogenetic responses were achieved in patients in all phases of CML and in patients with Ph+ ALL (Additional file 1, Table S2) [42-49]. The responses observed in these studies were durable; among patients with CP CML who achieved a MCyR in the Phase II START C trial, 88% had maintained their response at 24-months follow-up [50]. Furthermore, responses were achieved across all BCR-ABL mutations, except T315I. These studies led to the approval of dasatinib for these patients [42-44].

START-R was a randomized, comparative trial of dasatinib 70 mg twice daily versus high-dose imatinib (800 mg) in patients with imatinib-resistant CP CML [49]. Patients receiving dasatinib had a significantly greater rate of hematologic and cytogenetic responses than patients receiving high-dose imatinib (Additional file 1, Table S2). Dasatinib was also superior to high-dose imatinib with regard to progression-free survival (PFS) (hazard ratio: 0.14; *P *< .001).

#### Phase III dose optimization studies

Although dasatinib 70 mg twice daily was the chosen dose in the Phase II START program, a Phase I study showed similar efficacy between once-daily and twice-daily treatment schedules, with significant hematologic and cytogenetic responses achieved with both regimens [34]. Long-term follow-up of this Phase I study showed that pleural effusions were less frequent with a once-daily schedule than with twice-daily dosing [51]. In addition, as a result of dose reductions, the median total daily dose across the Phase II program in patients with CP CML was close to 100 mg/day [43,48]. In light of these findings, two open-label, Phase III trials have been conducted to evaluate the optimal dosing regimen of dasatinib in CML and Ph+ ALL.

In the CA180-034 study [44,52], patients with CP CML who were resistant to or intolerant of imatinib (N = 662) were randomized to dasatinib 100 mg once daily, 50 mg twice daily, 140 mg once daily, or 70 mg twice daily. Marked and similar hematologic and cytogenetic efficacy was seen across all four dasatinib arms in the CA180-034 trial. However, there was a difference in safety profiles. Dasatinib 100 mg once daily was associated with a reduced incidence of cytopenia and pleural effusions compared with the 70 mg twice-daily arm and with the lowest incidence of treatment interruption and discontinuation compared with all the other arms. The rate of discontinuation due to toxicity was lower compared with 70 mg twice daily (6% vs 15%), suggesting that once-daily dasatinib is better tolerated than a twice-daily schedule. The rates of treatment interruption and dose reduction for any reason were also lower with 100 mg dasatinib once daily compared with 70 mg twice daily (58% vs 71% and 33% vs 57%, respectively). The results of this study demonstrate that a 100 mg once-daily dose of dasatinib offers the most favorable overall benefit/risk assessment in CP CML, and have prompted a change in the recommended starting dose of dasatinib to this regimen [35]. It should be noted that the recommended starting dose remains 70 mg twice daily for patients with advanced phase disease [35].

### Adverse events and management recommendations

The most common drug-related serious toxicities in dasatinib clinical trials were fluid retention (8%), pleural effusion (5%), diarrhea (3%), skin rash (1%), headache (1%), hemorrhage (6%), fatigue (2%), nausea (1%), and dyspnea (4%). Discontinuation rates as a result of toxicities were 9%, 10%, 15%, and 8% for patients with CP, AP, and BP CML, and Ph+ ALL, respectively [35]. Grade 3/4 dasatinib-related AEs were generally comparable in all phases of CML. However, fluid retention/pleural effusion, hemorrhage, and febrile neutropenia were of greater frequency in advanced CML [42-49]. Of particular note was the lack of cross-intolerance with imatinib; there was no recurrence of AEs associated with imatinib intolerance. Imatinib intolerance was defined as the occurrence of at least a grade 3 nonhematologic or grade 4 hematologic toxicity that persisted for more than 7 days as a result of imatinib therapy [44]. Furthermore, the tolerability profile of dasatinib was comparable between imatinib-resistant and -intolerant patients [42].

As cytopenias (eg, neutropenia and thrombocytopenia) and fluid retention are commonly associated with dasatinib treatment, patients should be monitored closely for these AEs. Complete blood counts should be performed weekly for the detection of cytopenias, especially during the first two months following initiation of therapy. Adjustment of the dose of dasatinib may be advisable in order to manage cytopenias (Additional file 1, Table S3) [35]. Doses can be reduced from 140 to 100 mg/day or from 100 to 80 mg/day to allow continuation of dasatinib treatment. Patients with advanced phase disease may have disease-related cytopenias. Therefore, treatment discontinuation or interruption is not recommended due to the poor prognosis of these patients [11]. Myeloid growth factors and/or platelet transfusions may help to lessen the risks of complications associated with neutropenia and thrombocytopenia, respectively, and reduce the need for dose modification [11,53,54].

Patients should be monitored closely for the signs and symptoms of fluid retention, such as pitting edema, shortness of breath, and rapid weight gain. Patients exhibiting these symptoms should undergo radiographic studies to promptly detect pleural effusions. Fluid retention can be effectively managed with supportive care (diuretics, steroids) and dose interruption [11,55]. In severe cases of fluid retention, the drug should be held until symptoms improve to grade 1 or better (mild or asymptomatic), and then the dose of dasatinib should be reduced from 140 to 100 mg/day or from 100 to 80 mg/day. In clinical trials, early identification, temporary interruption of treatment, diuretic and/or pulse steroid usage, and subsequent dasatinib dose reduction led to resolution of adverse event-related symptoms in all cases [49,56].

QT prolongation is a rare but severe AE that should be monitored during dasatinib therapy. All patients should be screened for risks of QT prolongation such as hypokalemia, hypomagnesemia, congenital long QT syndrome, and concurrent mediations that can lead to QT prolongation (Additional file 1, Table S1). All electrolyte abnormalities should be corrected prior to initiation of dasatinib. Other grade 3/4 laboratory abnormalities such as hypocalcemia and hypophosphatemia have been reported with dasatinib [35].

In clinical studies, the majority of nonhematologic AEs were mild to moderate (grades 1/2) in severity and resolved spontaneously without the need for dose modification. Interventions for managing grade 3 nonhematologic AEs are shown in Additional file 1, Table S4 [11]. For grade 3 nonhematologic AEs that do not respond to symptomatic measures, dasatinib should be withheld until the AE resolves to grade 1 or better (asymptomatic). Dasatinib treatment can then be resumed, and lower doses should be considered [11].

## Summary

Dasatinib represents a promising treatment option for patients in all phases of CML and for patients with Ph+ ALL for whom treatment with imatinib has failed. Resistance to imatinib is a significant clinical issue in the treatment of CML and Ph+ ALL; however, treatment with dasatinib can overcome intrinsic or acquired resistance to imatinib.

In clinical trials, hematologic and cytogenetic responses to dasatinib were durable and were achieved in patients in all phases of CML as well as in patients with Ph+ ALL [42-49]. Compared with high-dose imatinib (800 mg), dasatinib 70 mg twice daily demonstrated a significantly greater rate of hematologic and cytogenetic responses in patients with imatinib-resistant CP CML [49]. A Phase III dose optimization study revealed that the safety profile of dasatinib was improved and efficacy maintained when dasatinib was administered to patients with CP CML on a 100 mg once-daily schedule [44]. Furthermore, while acknowledging the potential safety benefits of dasatinib 140 mg once daily, another Phase III trial demonstrated that dasatinib 70 mg administered twice daily to patients with advanced CML or Ph+ ALL resulted in a more durable response and longer PFS compared with once-daily dosing [57].

AEs associated with dasatinib treatment are typically mild to moderate, and most can be resolved with temporary withdrawal of dasatinib and/or dose adjustments. Patients should be monitored for AEs commonly associated with dasatinib, such as cytopenias and fluid retention. Adjusting the dose of dasatinib from 140 to 100 mg/day or from 100 to 80 mg/day may allow continuation of dasatinib in some cases.

In patients with CP CML, dasatinib 100 mg once daily improves the safety profile and compliance while maintaining efficacy compared with the 70 mg twice-daily schedule. Because of these findings, 100 mg dasatinib once daily is now approved for patients with CP CML. In patients with AP CML, BP CML, or Ph+ ALL, the recommended dose remains 70 mg dasatinib administered twice daily.

## Competing interests

SFW has received honoraria for serving as a consultant at advisory boards for BMS.

## Supplementary Material

Additional File 1 **Wong Tables 1–4.** The data provided in these tables are varied: Table 1 describes agents that may interact with dasatinib; Table 2 describes the efficacy data for dasatinib from a large phase II trial program; Table 3 and 4 provide management recommendations for cytopenias and non-hematologic AEs, respectively. Click here for file
