# Supplementary material for: New dosing schedules of dasatinib for CML and adverse event management
Source: J Hematol Oncol. 2009 Feb 23;2:10. doi: 10.1186/1756-8722-2-10 (PMC2649947; doi:10.1186/1756-8722-2-10)
Supplement: Additional File 1 — Wong Tables 1–4. The data provided in these tables are varied: Table 1 describes agents that may interact with dasatinib; Table 2 describes the efficacy data for dasatinib from a large phase II trial program; Table 3 and 4 provide management recommendations for cytopenias and non-hematologic AEs, respectively. [file 1756-8722-2-10-S1.doc]

**Table S1.** Overview of drug-drug interactions associated with dasatinib [11, 34,38]

|  | **CYP3A4 Inhibitors** | **CYP3A4 Inducers** | **CYP3A4 Substrates** | **QT Prolongation Medications** | **Antacids and Proton Pump Inhibitors** |
| --- | --- | --- | --- | --- | --- |
| Agent | Ketoconazole, itraconazole, erythromycin, clarithromycin, ciprofloxacin, ritonavir, atazanavir, indinavir, nefazodone, nelfinavir, saquinavir, telithromycin, norfloxacin, diltiazem, fluvoxamine, cimetidine, fluconazole, grapefruit juice | Dexamethasone, phenytoin, carbamazepine, rifampin, phenobarbital, efavirenz, nevirapine barbiturates, glucocorticoids, rifabutin, famotidine, *Hypericum perforatum* (also known as St. John’s Wort), rifampicin | Simvastatin, cyclosporine  Alfentanil , asternizole, terfenadine, fentanyl, quinidine, sirolimus, tacrolimus, ergot alkaloids | Quinidine, procainamide, amiodarone, erythromycins, clarithromycin, chlorpromazinem haloperidol, thioridazine, droperidol, methadone , arsenic trioxide, domperidone, halofantrine, pentamidine | Antacids  H_2_ blockers  Proton pump inhibitors (PPI) |
| Description | Increase plasma level of dasatinib | Decrease plasma level of dasatinib | Increase substrate level | Increased risk of QT prolongation | Alteration in the AUC of dasatinib |
| Action | Monitor closely for toxicity; consider dasatinib dose reduction of 20-40 mg daily | Use CYP3A4 inducers with less enzyme induction potential if possible; dose increases of dasatinib in 20 mg increments are recommended. | Monitor closely for toxicity of the substrates | Monitor closely for QT prolongation with ECG; check serum electrolytes (potassium &magnesium) and correct prior to initation of dasatinib and during therapy. | Antacids: administer 2 hours apart  H2 blockers & PPI : avoid if possible, substitute with antacids |

**Table S2.** Response rates in phase II START programs of dasatinib [39, 42-48]

| **Study**^a^ | **START-C** | **START-A** | **START-B** | **START-L** | **START-R** | |
| --- | --- | --- | --- | --- | --- | --- |
|  |  |  |  |  | **Dasatinib** | **High-dose imatinib (800 mg/day)** |
| N | 387 | 174 | 109 | 48 | 101 | 49 |
| Disease stage | CP | AP | MBP | LBP | CP | CP |
| Median follow-up, months | 15.2 | 14.1 | 20^a^ | 20^a^ | 15 | 15 |
| Response (%) |  |  |  |  |  |  |
| CHR | 91 | 45 | 27 | 29 | 93 | 82^b^ |
| MCyR | 59 | 39 | 33 | 52 | 52 | 33^c^ |
| CCyR | 49 | 32 | 26 | 46 | 40 | 16^d^ |

AP, accelerated phase CML; CCyR, complete cytogenetic response; CHR, complete hematologic response; CP, chronic phase CML; CML, chronic myelogenous leukemia; LBP, lymphoid blast phase CML; MBP, myeloid blast phase CML; MCyR, major cytogenetic response

^a^70 mg twice daily dasatinib was used in all studies and the maximum duration of follow-up is reported.

^b^*P* = .034

^c^*P* = .023

^d^*P* = .004

**Table S3.** Management recommendations of neutropenia and thrombocytopenia associated with dasatinib [34]

| **CP CML** (starting dose 100 mg QD) | Monitoring:  Complete blood counts (CBC with differential) weekly for first 2 months and then monthly thereafter, or as clinically indicated | ANC <0.5 x 10^9^/L and/or platelets <50 x 10^9^/L | 1. Stop dasatinib until ANC ≥1.0 x 10^9^/L and platelets ≥50 x 10^9^/L 2. Resume treatment at the original starting dose if recovery occurs in ≤ 7 days 3. If platelets <25 x 10^9^/L and/or recurrence of ANC <0.5 x 10^9^/L for >7 days, repeat step 1 and resume dasatinib at a reduced dose of 80 mg QD (second episode) or discontinue (third episode) |
| --- | --- | --- | --- |
| **AP CML, BP CML and Ph+ ALL** (starting dose 70 mg BID) | Monitoring  Complete blood counts (CBC with differential) as clinically indicated | ANC <0.5 x 10^9^/L and/or platelets <10 x 10^9^/L | 1. Determine whether cytopenia is related to leukemia (using marrow aspirate and/or biopsy) 2. If cytopenia is unrelated to leukemia, stop dasatinib until ANC ≥1.0 x 10^9^/L and platelets ≥20 x 10^9^/L and resume at the original starting dose 3. If recurrence of cytopenia, repeat step 1 and resume dasatinib at a reduced dose of 50 mg BID (second episode) or 40 mg BID (third episode) 4. If cytopenia is related to leukemia, consider dose escalation to 100 mg BID |

ANC = absolute neutrophil count; AP, accelerated phase; BID = twice daily; BP, blast phase; CML, chronic myelogenous leukemia; CP, chronic phase; Ph+ ALL, Philadelphia chromosome positive acute lymphoblastic leukemia; QD = once daily

**Table S4.** Management recommendations for severe (Grade 3/4) nonhematologic adverse events associated with dasatinib [11]

| **Adverse Event** | **Incidence^a,b^** | **Signs and Symptoms** | **Monitoring** | **Intervention^b,c^** |
| --- | --- | --- | --- | --- |
| Pleural effusions | 22% (all grades); 5% (grades 3/4) | Dyspnea  Dry cough  Rapid weight gain | Evaluate by chest X-ray upon presentation of signs and symptoms | Grade 3: (*Definition: Symptomatic and supplemental oxygen, >2 therapeutic thoracenteses, tube drainage, or pleurodesis indicated*)  Interupt dasatinib treatment. Initiate oxygen and diuretics. For significant symptoms use a short course of steroids (prednisone 20 mg/day x 3).  Grade 4: (*Definition: Life-threatening [e.g.,causing hemodynamic instability or ventilator support*])  Hold dasatinib until grade 1 or better, then consider resuming dose at a reduced dose level.^d^ Supportive care with diruetics, oxygen, and systemic steroids. |
| Headache | 24% (all grades); 1% (grades 3/4) | Pain in the head, blurred vision, nausea, vomiting, hearing impairment, irritability, malaise | Self-monitoring and rating of headache intensity | Grade 3: (*Definition:Severe pain; pain or analgesics severely interfering with ADL*)  Supportive care with analgesics.  Grade 4: (*Definition: Disabling*)  Hold dasatinib until grade 1 or better, then consider resuming dose at a reduced dose level.^d^ Supportive care with analgesics |
| Nausea | 22% (all grades); 1% (grades 3/4) | Nausea, belching, heartburn, cramping, abdominal distention | NA | Grade 3: (*Definition: Inadequate oral caloric or fluid intake; IV fluids, tubefeedings, or TPN indicated ≥24 hrs*)  Hold dasatinib until grade 1 or better. Supportive care with antiemetic(s), fluid and electrolytes as needed.  Grade 4: (*Definition: Life-threatening consequence*)  Hold dasatinib until grade 1 or better, then consider resuming dose at a reduced dose level.^d^ Supportive care with antiemetic(s), fluid and electrolytes as needed. |
| Diarrhea | 31% (all grades); 3% (grades 3/4) | Frequent, loose, watery stools, abdominal cramps, bloating, may be preceded by nausea and vomiting | NA | Grade 3: (*Definition: Increase of ≥7 stools per day over baseline; incontinence; IV fluids ≥24 hrs; hospitalization; severe increase in ostomy output compared to baseline; interfering with ADL*)  Hold dasatinib. Supportive care with anti-diarrheals, fluid and electrolytes replacement.  Grade 4: (*Definition: Life-threatening consequences (e.g. hemodynamic collapse*)  Hold dasatinib until grade 1 or better, then consider resuming dose at a reduced dose level.^d^ Supportive care with anti-diarrheals, fluid and electrolytes relplacement. |
| Gastrointestinal bleeding (GI Hemorrhage) | 3% (all grades); 1% (grade 3/4) | Blood in the stool; diarrhea; signs and symptoms of anemia due to blood loss | Platelet counts | Grade 3 (*Definition:Transfusion, interventional radiology, endoscopic, or operative intervention indicated*) and Grade 4 (*Definition: Life-threatening consequences; major urgent intervention indicated*)  Modify dasatinib dose according to recommendations for thrombocytopenia (Table 4). For severe GI hemorrhages, dasatinib treatment must be interrupted and blood product transfusion may be indicated. Dasatinib may be reintroduced with caution at a lower dose level. |
| Rash | 22% (all grades); 1% (grades 3/4) | Itching, pain, swelling, erythemapustular lesions | NA | Symptomatic control with Regenecare™ for itch and pain, with or without minocycline for inflammation. Topical or systemic steroids may be considered in severe cases. Dose reduction, interruption or discontinuation of dasatinib may be indicated depending on presenting conditions of the skin rash. |

^a^Incidence across all clinical trials of dasatinib

^b^Grading system per the National Cancer Institute Common Terminology Criteria for Adverse Events Version 3.0 (NCI CTCAE v3.0): grade 1 = mild, grade 2 = moderate; grade 3 = severe; grade 4 = life-threatening/disabling

^c^Interventions recommended by the National Comprehensive Cancer Network (NCCN)

^d^Dose reductions: Chronic phase, 100 mg once daily 🡪 80 mg once daily (-1 level); Advanced phase, 70 mg twice daily 🡪 50 mg twice daily (-1 level) 🡪 40 mg twice daily (-2 level)
